# Dual-task training and cognitive performance in individuals with coronary artery disease and/or heart failure: a systematic review

**DOI:** 10.3389/fcvm.2025.1462385

**Published:** 2025-03-06

**Authors:** Talita Cezareti, Wallace Machado Magalhães de Souza, Andrea Camaz Deslandes, Tereza Cristina Felippe Guimarães, Daniel Arthur Barata Kasal, Luiz Fernando Rodrigues Junior, Mauro Felippe Felix Mediano

**Affiliations:** ^1^ Department of Research and Education, National Institute of Cardiology, Rio de Janeiro, Brazil; ^2^ Center for Cardiology and Exercise, Aloysio de Castro State Institute of Cardiology, Rio de Janeiro, Brazil; ^3^ Institute of Psychiatry, Federal University of Rio de Janeiro, Rio de Janeiro, Brazil; ^4^ Department of Physiological Sciences, Biomedical Institute, Federal University of the State of Rio de Janeiro, Rio de Janeiro, Brazil; ^5^ Evandro Chagas National Institute of Infectious Diseases, Oswaldo Cruz Foundation, Rio de Janeiro, Brazil

**Keywords:** coronary artery disease, heart failure, myocardial ischemia, multitasking behavior, dual task, cognition, cognitive performance

## Abstract

**Introduction:**

Dual-task training (DTT) emerged as a promising intervention strategy to improve cognition in individuals with cardiovascular diseases (CVDs). The aim of this study is to describe the literature on the relationship between motor-cognitive DTT and cognitive performance (CP) in individuals with coronary artery disease (CAD) and/or heart failure (HF).

**Method:**

This systematic review includes intervention and observational studies that assessed motor-cognitive DTT on CP in individuals with CAD and/or HF. Searches were performed in the MEDLINE/Pubmed, Scielo, Lilacs, PEDro, and EMBASE databases. Methodological quality was assessed using the PEDro and ROBII scales for intervention studies and the Newcastle-Ottawa Scale for observational studies. The certainty of evidence was assessed using Grading of Recommendations, Assessment, Development and Evaluation (GRADE) approach**.**

**Results:**

A total of 2,098 articles were retrieved, and 21 articles were selected for full reading. Among these, 16 were excluded according to pre-specified exclusion criteria, resulting in five studies conducted between 2018 and 2022, conducted in three countries (United States, Portugal, and Russia). The studies included 228 individuals, comprising one study with HF participants, one including women with CAD, two including individuals that underwent myocardial revascularization, and one with patients with CAD enrolled in a phase 2 of cardiac rehabilitation program. Each study used different combinations of motor and cognitive tasks, conducted sequentially (*n* = 2 studies) or simultaneously (*n* = 3 studies), with one study using virtual training. The overall certainty of evidence for CP was low according to GRADE approach. Reduction in postoperative cognitive dysfunction after myocardial revascularization was observed in two studies. Moreover, the results indicate that DTT may have a positive impact on memory, selective attention, and conflict resolution capacity.

**Conclusion:**

The studies reviewed indicate motor-cognitive DTT as a potential approach to improve CP in individuals with CAD and/or HF.

**Systematic Review Registration:**

www.crd.york.ac.uk/prospero/display_record.php?ID=CRD4202341516, identifier (CRD 4202341516).

## Introduction

Cardiovascular diseases (CVDs) represent a substantial global health burden, contributing to a high morbidity and mortality rates worldwide (1). Individuals with CVDs often experience cognitive impairment (CI), which can significantly impact their cognitive performance (CP) and overall well-being (2). CP encompasses information processing, intelligence, reasoning, as well as language development and memory. The mechanisms underlying cognitive decline include chronic inflammation and cerebral hypoperfusion, which are common pathways in CVD conditions such as coronary artery disease (CAD) and heart failure (HF) (3). Moreover, cardiovascular risk factors are closely linked with increased risk of dementia. A systematic review with meta-analysis that included 16 studies on CAD (involving 1,309,483 individuals) and 7 studies on HF (involving 1,958,702 individuals) found that CAD was associated with a 27% higher risk of dementia, while HF was linked to a 60% higher risk (4). Some of the risk factors for dementia are common to CVDs, such as low socioeconomic status, comorbidities (e.g., diabetes, hypertension, obesity), physical inactivity, smoking, depression, and lack of social interaction, underscoring the complex interplay between cardiovascular health and cognitive function (5). In this setting, comprehensive approaches integrating both physical and cognitive training may represent an important strategy for prevention and treatment of CI in individuals with CVDs (6).

The performance of activities of daily living (ADLs) often involves combined tasks that integrate motor and cognitive aspects, which can occur simultaneously or sequentially. Dual-task training (DTT), a strategy that combines two different tasks, has gained attention as a promising intervention to improve both physical and cognitive function (7–11). Different definitions and approaches have been proposed for DTT, primarily focusing on the combination of physical training with cognitive stimulation (motor-cognitive DTT), that potentially surpasses the benefits obtained from physical or cognitive training alone (12). The mechanisms underlying DTT are not yet fully understood, but two theories are frequently discussed in the literature. The serial bottleneck model suggests that the brain has a limited capacity to process simultaneous information, similar to a bottleneck effect. In contrast, the capacity-sharing model proposes that while multiple tasks can be processed in parallel, central processing capacity has a finite limit. Therefore, considering that DTT involves the combination of motor and cognitive tasks during physical exercise, activities such as virtual reality (VR) training can be considered a form of DTT, as it simultaneously engages both motor and cognitive demands (13–15). Despite the increasing number of studies about motor-cognitive DTT over the last recent years, its application in individuals with CVDs, particularly those at high risk of CI such as those with CAD and HF, remains scarce. Therefore, the aim of this study was to describe the literature on the relationship between motor-cognitive DTT and CP in individuals with CAD and/or HF.

## Methods

### Study design

This systematic review of the literature included interventional and observational studies, published in any language, involving individuals with coronary artery disease (CAD) and/or heart failure (HF) who were exposed to dual-task training (DTT), with CP as the outcome. The protocol of this systematic review was registered in PROSPERO and approved on 04/28/2023, under registration number CRD 4202341516. This systematic review adheres to the Preferred Reporting Items for Systematic Reviews and Meta-Analysis (PRISMA) guidelines (16). We excluded studies enrolling individuals with neurological and/or psychiatric disorders limiting cognitive function assessment, protocol studies, and gray literature, such as technical reports, theses and dissertations, government documents, patents, manuals, and conferences abstracts.

The selected studies primarily centered on motor-cognitive DTT, conducted either simultaneously or sequentially. Physical training was recognized as interventions that consider specific prescription parameters such as frequency, intensity, type of exercise, or duration. Cognitive training encompassed interventions aimed at targeting single or multiple domains of cognitive function or intending to enhance CP.

### Search strategy

The PICOS strategy was used to formulate the research question for this systematic review as follows: (P) Population: individuals with CAD and/or HF; (I) Intervention: motor-cognitive DTT; (C) Comparison: any other comparator; (O) Outcomes: CP; (S) Study design: observational and intervention studies.

The search was conducted in the following databases: Medline/Pubmed, PEDro, Embase/Elsevier, Scielo, and Lilacs, with the search cutoff date set at June 11, 2023. The descriptors used can be found in the Supplementary File. In addition, an active search was conducted, which included email inquiries to researchers and a detailed examination of the reference lists of selected studies and systematic reviews on related topics identified during our research.

Data extraction was conducted by two independent blinded researchers (TC and WMMS). Rayyan (17) and Microsoft Excel were used to support selection and data extraction tables, containing information about the selected articles (author, year of publication, title, digital object identifier, study type, population, sample size, outcome, intervention, and main results). Artificial intelligence tools (ChatGPT and Google Translator) were used to translate manuscripts published in languages other than English.

### Methodological quality assessment

The methodological quality of intervention studies was assessed using the PEDro (18) scale and the Rob II scale (19). The PEDro scale is designed for intervention studies and consists of 11 items, with only 10 items being considered for scoring (excluding the question about external validity) (18). Studies with scores <4 were considered “poor”, those between 4 and 5 were considered “fair”, those between 6 and 8 were considered “good”, and those scoring 9–10 were considered “excellent” in terms of quality (18). The Rob II scale assesses bias risk in randomized trials, structured into domains focusing on trial design, conduct, and reporting. The Rob II scale uses signaling questions to determine risk of bias within each domain, yielding judgments of “low”, “some concerns”, or “high” risk of bias (19).

The methodological quality of observational studies was evaluated using Newcastle–Ottawa Scale (NOS), designed for case-control and cohort studies. The NOS assesses participant selection, comparability between groups, and outcome assessment, with a maximum score of 9 points (20).

### Certainty of evidence assessment

The quality of evidence from the included studies was evaluated using the Grading of Recommendations, Assessment, Development, and Evaluation (GRADE) approach. The GRADE system evaluates eight domains: (i) risk of bias, (ii) inconsistency of results, (iii) indirectness, (iv) imprecision, (v) publication bias, (vi) magnitude of effect, (vii) plausible confounding, and (viii) dose-response gradient. The overall certainty of the evidence is categorized as “very low”, “low” (further research is very likely to alter the effect estimate), “moderate” (further research may change the effect estimate), and “high” (further research is unlikely to significantly impact the effect estimate) (21).

### Effect measures

Effect measures considered both difference and ratio measurements. Difference measures were used to identify absolute differences between two groups or conditions, whether in terms of means, proportions, or other metrics. The ratio measures were used to identify the probability of event occurring in the exposed group in comparison to non-exposed group, and included relative risk, prevalence ratio, or odds ratio.

### Vote counting

The vote counting (22) was used to facilitate data synthesis when a meta-analysis was not possible. It is a simple method for synthesizing evidence from multiple assessments and involves comparing the number of positive studies (studies that show benefit) with the number of null (studies that show no effect) or negative studies (studies that show harm). The vote counting does not take into account the quality of the studies, the size of the samples nor the size of the effect (22).

## Results

**Figure 1:**
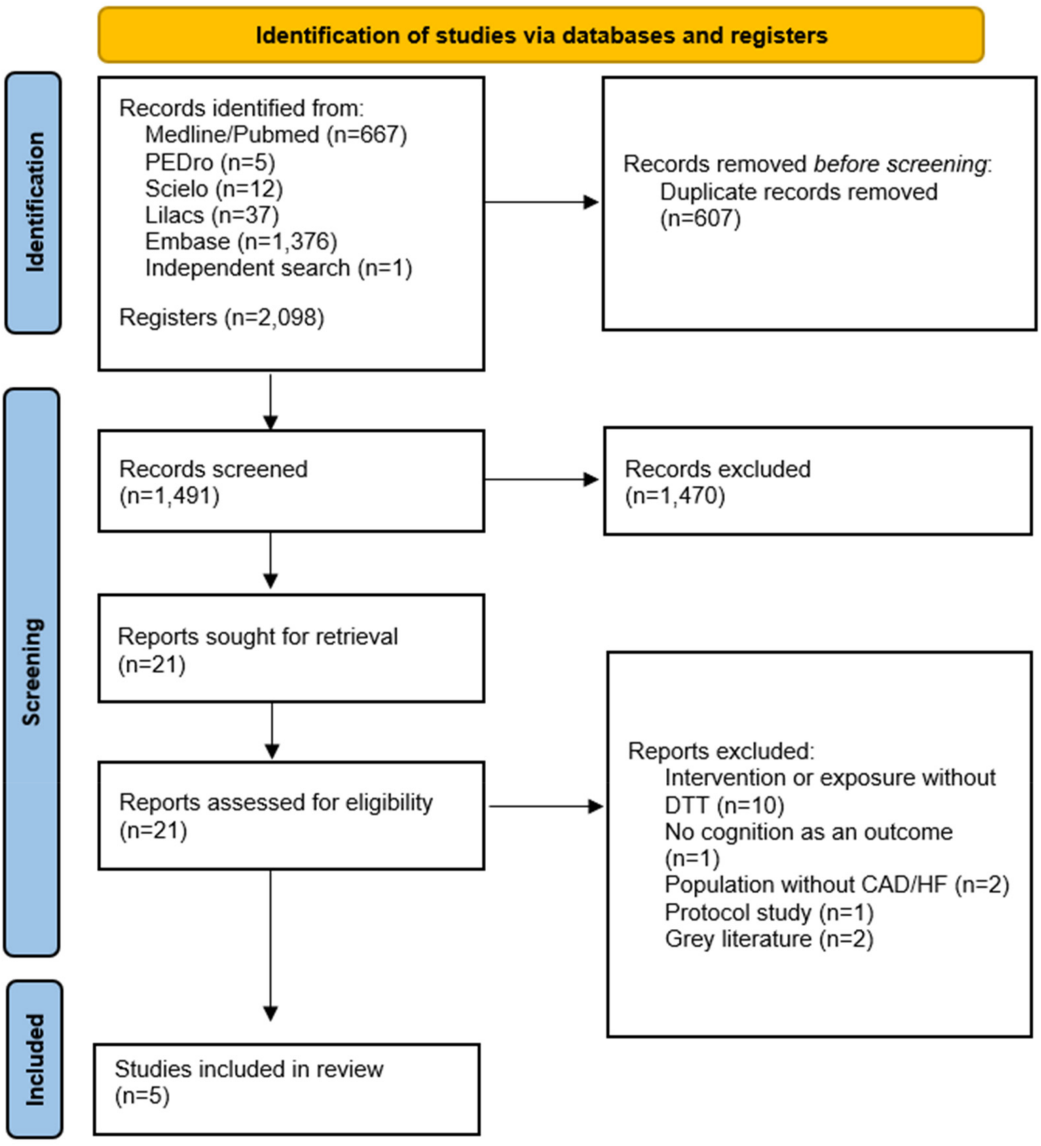
Flowchart of articles retrieved in the search and selection.

Database searches yielded 2,098 articles, comprising 667 from Medline/Pubmed, 12 from Scientific Electronic Library Online (SciELO), 37 from Lilacs, from PEDro, and 1,376 from EMBASE. Out of these, 607 were identified as duplicates across databases and removed from the study. Subsequently, 1,470 were excluded after title and abstract screening for not meeting inclusion criteria. Finally, 21 articles were selected for full reading, out of which 16 were excluded for not meeting the eligibility criteria. The reasons for exclusion included 10 articles due to intervention or exposure without a dual motor-cognitive task, two articles with population study without CAD and/or CI, one without cognition in the outcome, one protocol study article, and two articles from grey literature (abstract and congress protocol). Therefore, 5 studies were selected for the data extraction. Figure 1 shows the screening process selection.

The five included studies comprised a total of 228 patients with CVD. The studies were conducted in three countries (United States, Portugal, and Russia) and were published between 2018 and 2022. Three articles were published in English, and two articles were published in Russian. Four articles were intervention studies, and one observational study. Table 1 summarizes the data extracted from the selected articles. The frequency of interventions ranged from 3 to 7 times per week for 15–60 min each. The duration of interventions ranged from 1 to 24 weeks. Participants had a heart rate-based motor task intensity of 60%–70% in the inclusion studies. Furthermore, the two Russian studies did not describe the intensity of the intervention exercise.

**Table 1:** Main information of studies included in the review.

Author/year Doi	Country	Design	Sample characteristics	N	Outcome	Intervention/exposure	Cognitive assessment	Results
Vieira et al. (2018) (23) DOI:10.1080/17483107.2017.1297858	Portugal	Randomized clinical trial	Men who completed phase 2 of cardiac rehabilitation. Presence of CAD. Mean age: 57.7 years	33	Executive function, quality of life, and depression	Intervention divided into three groups: G1—cardiovascular rehabilitation program at home using virtual reality (Kinect); G2—same protocol as G1, but with instructions via a paper booklet; G3—usual care. Exercise protocol: warm-up; seven conditioning training exercises aimed at improving cardiorespiratory and muscular endurance and/or strength; and two flexibility exercises	Primary cognitive screening—MoCA; EF—trail making test, verbal digits test, and stroop test	DTT through virtual reality showed significant improvements in selective attention, conflict resolution, and quality of life. No significant differences in program adherence were found between groups 1 and 2. No significant differences were observed in depression, anxiety, and stress variables throughout the study
Gary et al. (2019) (24) DOI:10.1016/j.jagp.2019.01.211	United States	Randomized clinical trial	Patients with HF. Mean age: 61 years. 54% female	69	Memory, executive function, attention, processing speed, and reaction time	Intervention divided into three groups: G1—exercise (3× weekly walking for 24 weeks at 60%–70% of maximum heart rate). G2—Exercise+computerized cognitive training (same exercise protocol as group 1+Brain fitness. 40 1-h sessions for 8 weeks). G3— Control group with stretching, flexibility, and education (2–3 times weekly for 24 weeks)	Global cognition—MoCA; Verbal memory, visual memory, attention and processing speed—RBANS; Working memory—WAIS-IV; Reaction time—CalCap	Groups 1 and 2 showed improvement in verbal memory at 3 months compared to the control group. Group 2 demonstrated increased distance covered in the 6 MWT at 3 months compared to the control group. No significant differences were observed in depressive symptoms among the groups. Adherence to the walking program was 60%, with good adherence to cognitive exercises in Group 2
Halloway et al. (2021) (25) DOI:10.1123/jpah.2020-0206	United States	Quasi-experimental (single arm)	Women with CVD Mean age: 71.9 years 50% completed a university degree	10	Viability and acceptability of the MindMoves program: Changes in physical activity and cognitive function. Physical activity, Cardiorespiratory activity Fitness and cognitive function	Physical training with controlled aerobic activity monitored by FitBit. Goal of gradual increase to 3,000 daily steps and 150 active minutes per week. Cognitive training with BrainHQ (audio-based with 6 progressive and individualized exercises). Three 30-min sessions per week for 24 weeks	Cognitive function—NIH Toolbox® Adult Fluid Cognition Battery	Participants showed a significant increase in light, moderate to vigorous physical activity, and daily steps, but not in cardiorespiratory fitness and cognitive function. Program participation and participant satisfaction were high
Tarasova et al. (2021) (26) DOI: 10.17802/2306-1278-2021-10-3-15-25	Russia	Randomized clinical trial	Patients admitted for coronary artery bypass graft surgery with cardiopulmonary bypass Mean age: 56.4 years 70.8% men	48	Cognitive and postural functions and neurophysiological parameters in patients in the immediate postoperative period of myocardial revascularization	Postural and cognitive training. Postural training involved balancing on a computerized stable platform while concurrently performing one of three cognitive tasks: naming objects starting with a specific letter, subtracting sequentially 7 from 100, and naming uncommon uses for objects such as brick, newspaper, and ruler. DTT was conducted daily, starting 3–4 days after the procedure and continuing until hospital discharge, with 5–7 training sessions lasting 15–20 min each	Focused and distributed attention function: Burdon correction test. Neurophysiology: EEG	Cognitive dual-task training demonstrated a significant reduction in the risk of postoperative complications compared to the control group. Patients undergoing cognitive training showed a significant improvement in cognitive performance after surgery. Psychophysiological changes suggest trending improvements in neurodynamics and short-term memory in the cognitive training group. No significant differences were found in postoperative postural stability (stabilogram) and EEG frequency between the groups. There was a significant reduction in peak alpha frequency observed in both groups after surgery. The interaction between Group and Time was significant for theta-1 frequency, indicating specific changes associated with cognitive training
Trubnikova et al. (2022) (6) DOI: 10.15829/1728-8800-2022-3320	Russia	Prospective cohort	Patients admitted for coronary artery bypass graft surgery with cardiopulmonary bypass Mean age: 64 years 79% men	68	Cognition Neurophysiological parameters in patients in the immediate postoperative period of myocardial revascularization	Postural and cognitive training. Postural training involved balancing on a computerized stable platform while concurrently performing one of three cognitive tasks: naming objects starting with a specific letter, subtracting sequentially 7 from 100, and naming uncommon uses for objects such as brick, newspaper, and ruler. DTT was conducted daily, starting 3–4 days after the procedure and continuing until hospital discharge, with 5–7 training sessions lasting 15–20 min each	Cognitive functions: MoCA Neuropsychological tests. Neurophysiology: EEG	The incidence of postoperative cognitive dysfunction was lower in the cognitive training group (54.4%) compared to the comparison group (69.3%). Within the same group, the largest differences in the frequency of cognitive decline (20%) were observed in response time and error tasks, while memory tests showed a lower frequency of decline. The intervention group proved more effective in preventing cognitive decline in neurodynamic and short-term memory domains, being less effective in attention. Postoperative increase in the frontoparietal gradient in relation to theta1 rhythm. Dual-task cognitive training impacted the parieto-occipital areas of the brain, resulting in an increase in the frontoparietal gradient

IC, heart failure; EEG, electroencephalogram; NYHA, New York Heart Association; LVEF, Left ventricular ejection fraction; HFrEF, heart failure with reduced ejection fraction; HFmrEF, heart failure with mid-range ejection fraction; MoCA, Montreal cognitive assessment; RBANS, repeatable battery for the assessment of neuropsychological status; WAIS-IV, Wechsler adult intelligence scale—fourth edition; CalCap, California computerized assessment package; CVD, cardiovascular disease; DAC, coronary artery disease; 6MWT, 6-minute walk test; FitBit, wearables; DTT, dual task training; Brain-HK, online headquarters for brain working.

Vieira et al. (23) was a randomized clinical trial including men who completed a phase 2 of cardiac rehabilitation program. The study sample included 33 individuals hospitalized due to acute coronary syndrome, stable angina, or post-angioplasty. Participants were randomly assigned to three groups: Kinect virtual training group (*n* = 11; mean age 55.0 year), booklet-guided training group (*n* = 11; mean age 59.0 year), and a control group (*n* = 11; mean age 59.0 year). Participants were followed-up during 6 months and intervention groups exercised three times a week. The results showed that the Kinect virtual reality group demonstrated significant improvements in selective attention and conflict resolution ability compared to the control group.

Gary et al. (24) conducted a randomized clinical trial involving 69 outpatient patients with HF, with a mean age of 61 years. Most were classified as NYHA (New York Heart Association functional classification) class II (*n* = 38, 55%), with 53% (*n* = 36) having an implanted device such as a defibrillator or pacemaker. The mean left ventricular ejection fraction (LVEF) was 35%, ranging from 10% to 65%. Most of patients presented HF with reduced LVEF (*n* = 42, 61%), followed by HF with preserved LVEF (*n* = 19, 28%), and HF with intermediate LVEF (*n* = 8, 12%). The study aimed to evaluate the effectiveness of a combined program of aerobic exercise and cognitive training on memory compared to isolated exercise or usual care during 24 weeks. Participants were assigned to receive a usual care program (stretching and flexibility) (*n* = 19), exercise-only intervention (*n* = 29), or exercise + cognitive training (*n* = 21). Participants who underwent the combined program showed significant improvements in verbal memory and the distance covered in a 6-min walk compared to the other groups.

In a quasi-experimental single arm intervention study, Holloway et al. (25) included 10 women aged ≥65 years with CVD history (e.g., coronary artery disease), mean age of 71.9 years. The study aimed to assess the feasibility of a program combining physical activity and cognitive training during 24 weeks. Outcomes included feasibility, changes in physical activity patterns, and changes in CP assessed by the NIH Toolbox® Adult Fluid Cognition Battery, a tool designed to evaluate various aspects of fluid cognition in adults. The results showed that most participants adhered to the program, with a high level of satisfaction. There was an increase in physical activity levels (number of steps), but the effect on cardiorespiratory fitness and CP (fluid cognition) was small with a trend to improve.

In a study conducted by Tarasova et al. (26), 48 participants that underwent a myocardial revascularization surgery were enrolled in a randomized clinical trial. The intervention comprised 5–7 sessions, starting on the 3rd or 4th postoperative day. Of the 48 participants included in the study, 23 were randomly assigned to perform DTT, while 25 underwent motor task only (control group). Postoperative cognitive dysfunction was present in 39% of the DTT group compared to 64% in the control group (*p* = 0.08). Patients in the DTT group demonstrated improved cognitive status compared to preoperative levels (*p* = 0.01). Additionally, enhancements in short-term memory were observed in DTT group.

The last study, conducted by Trubnikova et al. (6), was a prospective cohort study involving 68 patients admitted for coronary artery bypass graft surgery. DTT exposure comprised 5–7 sessions, starting on the 3rd or 4th postoperative day. Early postoperative cognitive dysfunction was lower in DTT compared to data from non-training group observed in a previous study from the same group (54.4% vs. 69.3%; *p* = 0.04) (27). Furthermore, there were improvements in neurophysiological parameters assessed by electroencephalogram (EEG).

The studies presented a variety of DTT protocols, with two conducting sequential dual tasks and three employing simultaneous dual tasks, as outlined in Table 2.

**Table 2:** Synthesis chart of dual-task types and specified motor and cognitive demands.

Author/year	Dual-task type	Motor task	Cognitive task
Vieira et al. (2018) (23**)**	Simultaneous	Cardiac rehabilitation exercise protocol	Virtual training (Kinetic) following movements and commands
Gary et al. (2019) (24)	Sequential	Aerobic training (walking)	Brain fitness
Halloway et al. (2021) (25)	Sequential	Aerobic training (steps)	BrainHQ
Tarasova et al. (2021) (26)	Simultaneous	Postural training on stable platform	Naming objects, subtracting 7 from 100, and naming unusual uses
Trubnikova et al. (2022) **(**6**)**	Simultaneous	Postural training on stable platform	Naming objects, subtracting 7 from 100, and naming unusual uses

### Methodological quality assessment

**Figure 2:**
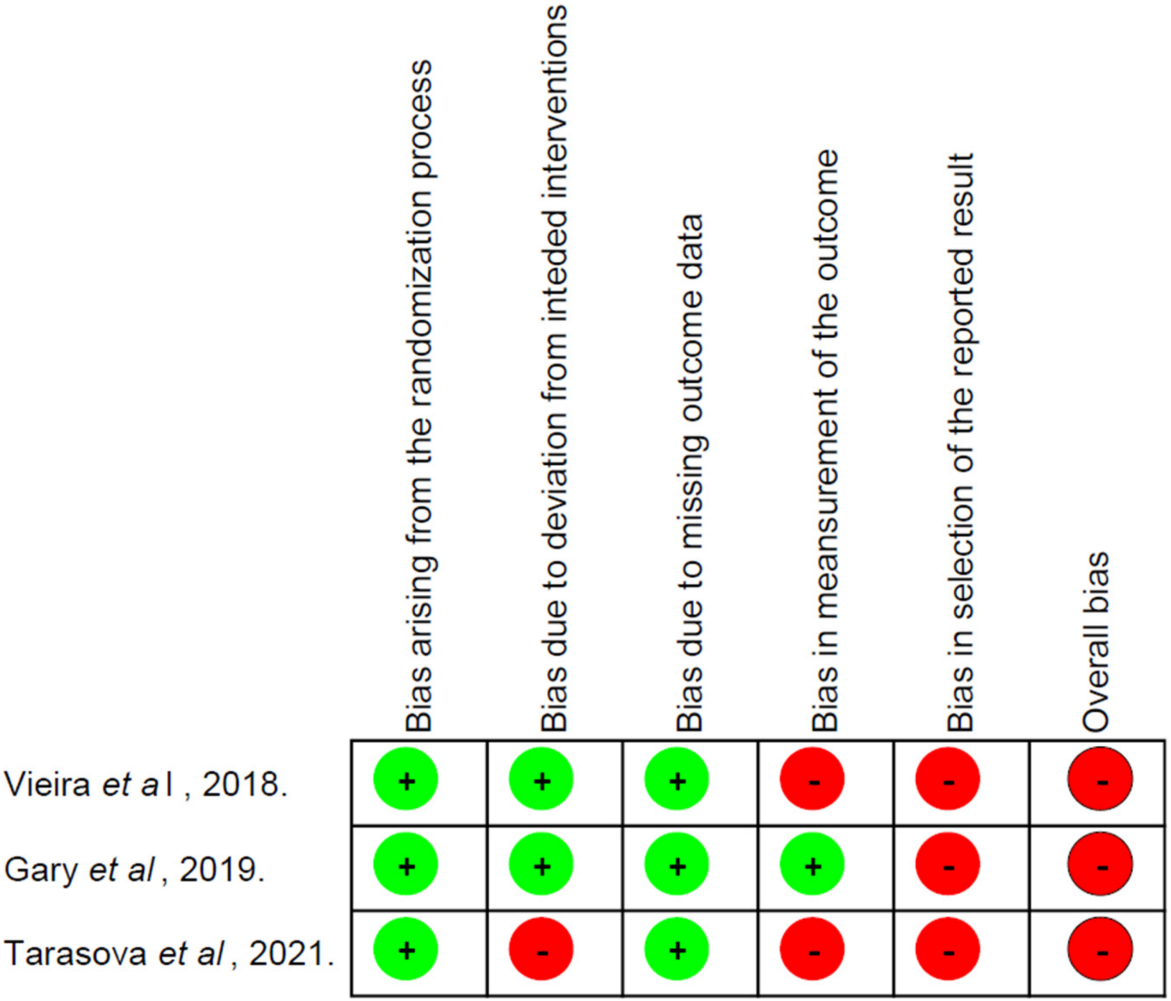
Methodological quality of intervention studies using RobII scale.

Figure 2; Tables 3, 4 illustrate the methodological evaluation of the studies. Among the four longitudinal studies, two (50%) were rated as having fair methodological quality, one (25%) as good, and one (25%) as poor, according to the PEDro scale. Of the three studies assessed using the ROBII scale, all were classified as having a high risk of bias. The apparent discrepancy between the PEDro scale score and the ROBII scale can be explained by the different criteria and thresholds each tool uses for assessing methodological quality. For example, in the case of Vieira et al. (23), while the study performed reasonably well on the PEDro scale, it was identified as having a high risk of bias in at least one critical domain according to the ROBII criteria, leading to its classification as having a high overall risk of bias. This underscores the more stringent nature of the ROBII tool in identifying specific sources of bias that may not be fully captured by the PEDro scale.

**Table 3:** Methodological quality of intervention studies using the PEDro scale.

PEDro scale criteria	Vieira et al. (2018) (23)	Gary et al. (2019) (24)	Halloway et al. (2021) (25)	Tarasova et al. (2021) (26)
1. Eligibility criteria	Yes	Yes	Yes	Yes
2. Random allocation	Yes	Yes	No	Yes
3. Concealed allocation	Yes	No	No	Yes
4. Baseline comparability	Yes	No	Yes	Yes
5. Subjects blinded	No	No	No	No
6. Therapists blinded	No	No	No	No
7. Assessors blinded	No	Yes	No	No
8. Adequate follow-up	Yes	No	Yes	No
9. Intention-to-treat	No	Yes	No	No
10. Between-group comparisons	Yes	Yes	Yes	Yes
11. Point estimates and variability	Yes	Yes	Yes	Yes
Total	6	5	4	5

**Table 4:** Methodological quality of the observational study using the Ottawa New Castle scale.

Newcastle-Ottawa scale	Study design	Selection	Comparability	Outcome	Total
Trubnikova et al. (2022) (6)	Prospective cohort	3	1	0	4

### Certainty of evidence

According to the GRADE criteria, the certainty of evidence for CP was initially rated as high, given that most of the included studies were randomized trials (3 out of 5; 60%). However, the evidence was downgraded by one level for risk of bias and by another level for indirectness of DTT intervention protocols and CP outcomes, resulting in an overall low certainty of evidence (Table 5). This indicates that further research is very likely to affect the estimate of the effect.

**Table 5:** Certainty of evidence using GRADE approach.

Outcome: cognitive performance							
Number of studies	Study design	Risk of bias	Inconsistency	Indirectness	Imprecision	Other considerations	Certainty
5	Randomized clinical trials (03); Quasi-experimental (01); Observational (01)	Serious	Not serious	Not serious	Serious	Not serious	⊕⊕○○ Low

### Vote counting

**Figure 3:**
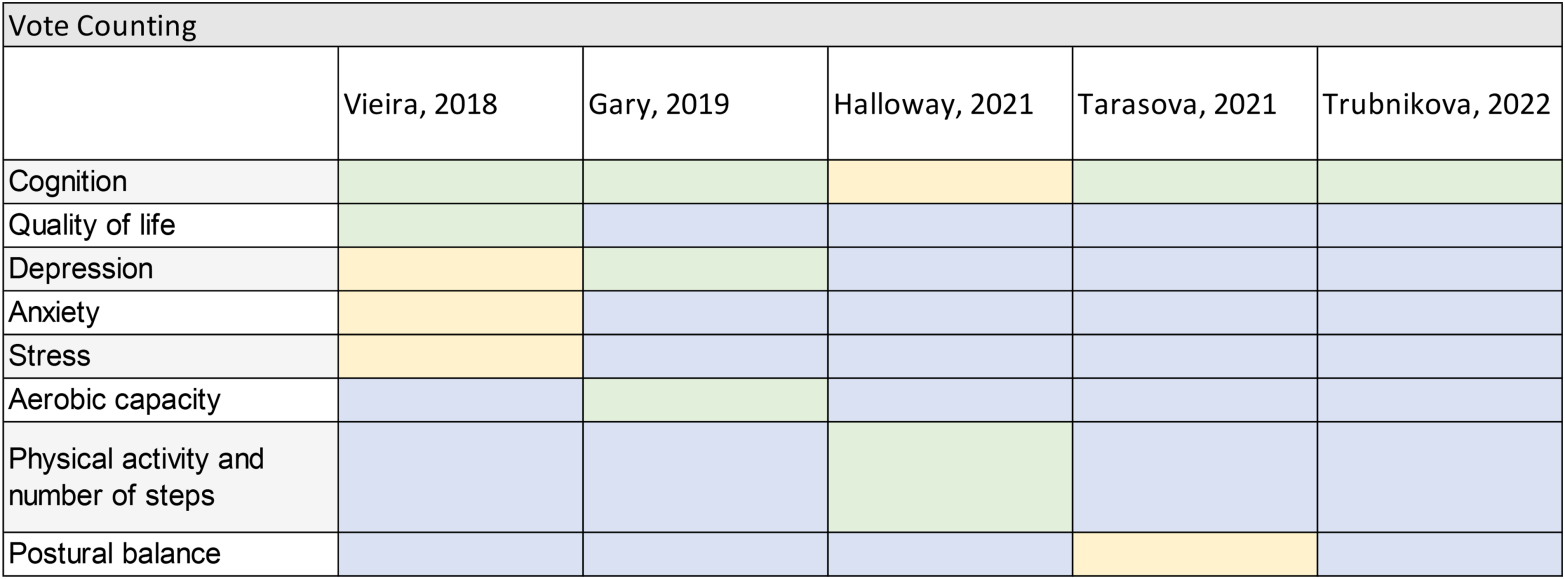
Summary of studies results. Green—significant positive effect; Yellow—no statistically significant effect on any direction (null result); Blue—outcome not evaluated; Red—significant negative effect (not observed).

Due to the heterogeneity of the studies, a meta-analysis was not feasible, therefore the comparison of studies results was conducted using a vote count method. In Figure 3, the articles are arranged by publication date, showing the outcomes evaluated in each study. Outcomes favoring DTT are highlighted in green, those without significant differences in yellow, and those not evaluated in blue. Four studies showed a positive trend for cognitive performance improvements with DTT, while one study did not find statistical significance, which was interpreted as a null result. However, this study found physical benefits, such as increased physical activity levels. No reported risks were associated with exposure among participants in any of the studies.

## Discussion

To the best of our knowledge, this is the first systematic review that addressed the relationship between DTT with CP in individuals with CVDs, with most of included studies demonstrating a positive trend towards cognitive performance improvement with DTT. Moreover, although the initial scientific evidence for potential benefits is still limited, none of the investigated studies reported any harmful effects of the intervention, further supporting its safety.

Previous literature has already demonstrated that DTT may play a positive role in enhancing cognition in individuals at a high risk of cognitive decline, such as older adults (28). For instance, Wollesen et al. (12) published a meta-analysis including 25 articles, concluding that DTT interventions showed improvements in domains related to global cognitive functions and inhibitory control. The same review also found that virtual training improved functions related to processing speed, attention, and inhibitory control. Similarly, Castalaño et al. (11) compared the effects of traditional resistance training alone vs. a DTT protocol comprising resistance training combined with a cognitive task on body composition, physical performance, cognitive function, and plasma BDNF levels in older adults. The study demonstrated improvements in cognitive function and BDNF levels only among those who underwent DTT. More recently, a systematic review with meta-analysis including 28 studies and 2,711 participants showed that DTT were associated with improved cognition in older individuals with mild cognitive impairment compared to single interventions (29).

In the Tarasova et al. study (26), we observed that DTT presented positive results for this category of patients. However, the authors recommend further research to explore the potential benefits of increasing the duration and intensity of DTT to enhance recovery outcomes and improve cognitive and walking performance in patients during the postoperative period of myocardial revascularization surgery. This recommendation highlights how early-stage studies of this intervention in specific populations, such as those in an intra-hospital setting, remain limited. According to the review by Herold et al. (8), the most effective interventions are those that simultaneously integrate physical and cognitive stimuli, known as simultaneous DTT. However, no previous studies have directly compared different DTT protocols on physical and cognitive performance. Therefore, further research is needed to compare various DTT protocols in the literature.

The results of our systematic review extend the knowledge of the potential benefits of DTT to individuals with CVDs, particularly those diagnosed with CAD and HF, which share several common pathophysiological pathways with CI (4, 30). Despite the literature evaluating the relationship between DTT and cognition in individuals with CVD is still scarce, the American Heart Association recently published a review article entitled “Cognitive Impairment in Patients With Cardiac Disease: Implications for Clinical Practice” that emphasizes the high prevalence of undetected CI in individuals with CVDs, and advocated for a systematic approach to enhance the identification and treatment of CI in this population (2).

The mechanisms by which DTT enhance cognition is still under investigation. Some of the possible neurobiological mechanisms that can explain cognitive improvements through DTT are neural plasticity, enhanced activation of neural networks, attentional and executive control enhancements, ultimately enhancing overall brain function, and influencing neurotransmitter levels and synaptic plasticity (31, 32). Moreover, motor-cognitive DTT may stimulate different areas of the brain, stimulating neural connectivity and plasticity (33, 34), improving cognitive flexibility, attentional control, and task-switching abilities, all of which are essential for daily living functioning (12). DTT may better simulates the complexity and dynamic nature of real-world activities, in which individuals often perform situations that require concurrent processing of motor and cognitive demands. Therefore, the importance of DTT relies in its ability to provide a comprehensive and functional approach to concomitantly improve both physical performance and cognition, with practical implications for daily living activities (34–37).

The reviewed studies differ in how they assess the severity of CVDs and also vary by age. The severity of CVD may influence CP, since severe CVD is associated with frailty, which in turn negatively affects cognition. Frailty, characterized by increased vulnerability to internal and external stressors due to decreased physiological reserves, may compromise CP, especially in patients with severe CVD. Furthermore, age is a significant risk factor for both CVD severity and cognitive impairment. Older individuals, particularly those over 75 years of age, often face coexisting geriatric syndromes that intensify the relationship between CVD and cognitive changes (38). There is a bidirectional relationship between cognitive impairment, frailty, and CVDs. Evidence of this correlation is the concept of cognitive frailty, which is characterized by the simultaneous presence of cognitive impairment, without evidence of dementia, and physical frailty, which results in decreased cognitive reserve (39). The interaction between age and CVD contributes to the increased risk of cognitive impairment, since common pathophysiological factors, such as inflammation and neurohormonal dysregulation, are present. Therefore, it is essential that future studies consider how CVD severity and age interact to better understand the impact on CP (38, 39).

In addition to its impact on cognition, DTT may offer additional health benefits. For instance, the study of Silveira et al. involving older individuals with CVDs indicated that poor DTT performance was associated with an increased risk of falls (40). Moreover, Park et al. addressed the clinical relevance of DTT for balance and functional efficiency in community-dwelling older adults with a history of falls. The authors concluded that 12 sessions of DTT were more effective in improving balance compared to conventional balance training (41). While a positive trend favoring DTT for CP is evident, cautious interpretation of the results is warranted, especially in studies using DTT immediately postoperative with extracorporeal circulation, which is typically linked to a greater decline in cognitive function during the postoperative period (42, 43).

Although the evidence supporting the benefits of DTT is still emerging, particularly in individuals with CVDs, the absence of adverse outcomes across all the studies included in our systematic review suggests that DTT can be a safe approach to improving cognitive and motor function. This makes it a promising intervention strategy for older individuals with CVDs. However, further studies are needed to investigate the potential risks of this intervention and more thoroughly confirm its safety.

### Limitations

This review has several important limitations. The studies included showed significant heterogeneity in participant characteristics, sample sizes, types of DTT interventions, CP and outcome measures. These variations hindered the feasibility of conducting a meta-analysis, which could have provided a more robust synthesis of the findings. Another potential limitation is the use of AI tools to translate non-English literature. To minimize translation errors, we used two different AI tools (ChatGPT and Google Translate) for the initial translations of the two Russian studies. Subsequently, we conducted a careful review and comparison of the translated texts to ensure that key information and nuances were accurately captured. Moreover, the exclusion of grey literature might have resulted in omission of relevant studies and data. However, incorporating grey literature poses challenges due to difficulties in assessing the quality of these studies.

### Future directions

Performing a systematic review with only a limited number of studies presents several challenges. The small sample size of available studies often leads to a lack of statistical power, making it difficult to draw definitive conclusions. Additionally, the heterogeneity in study designs, methodologies, and populations make the synthesis of results difficult, as comparisons between studies are not clear. This heterogeneity may result in a lack of consensus about the potential relationship of motor-cognitive DTT on cognitive outcomes, making it challenging to provide clear recommendations. Despite these difficulties, this systematic review is valuable in highlighting existing gaps in the literature and identifying areas for future research. With the aging of the population and the consequent increase in the prevalence of CVDs, it is crucial to explore preventive and therapeutic approaches that can directly impact CP. Furthermore, there is a need to evaluate different DTT protocols, particularly tailored for populations with specific clinical characteristics such as CAD and/or HF. Future randomized clinical trials and long-term follow-up studies are needed to assess the safety and sustainability of cognitive and physical improvements after DTT, determine whether the benefits observed in short-term interventions are maintained over time, investigate the potential for relapse in cognitive function, and develop strategies to prevent it following the completion of a DTT program.

## Conclusion

In conclusion, this review identified a limited number of articles addressing DTT in individuals with CAD or HF, suggesting a positive trend in improving CP. Importantly, no studies reported an increased risk of CI associated with DTT, and no adverse outcomes were noted among participants in any study. Based on these initial findings, we encourage the utilization of DTT in populations with CAD and/or HF to enhance CP.

## Data Availability

The original contributions presented in the study are included in the article/Supplementary Material, further inquiries can be directed to the corresponding author.
