# Supplementary material for: Dual-task training and cognitive performance in individuals with coronary artery disease and/or heart failure: a systematic review
Source: Front Cardiovasc Med. 2025 Mar 6;12:1462385. doi: 10.3389/fcvm.2025.1462385 (PMC11922836; doi:10.3389/fcvm.2025.1462385)
Supplement: Supplementary file 1 [file Datasheet1.pdf]

Supplementary Material

Descriptors used in literature search:

Population (cardiovascular disease, cardiology, heart disease, cardiac disease, coronary artery disease, coronary disease, angina pectoris, coronary artery bypass, heart failure, percutaneous coronary intervention, angioplasty balloon coronary, heart disease risk factors, cardiovascular surgical procedures, coronary heart disease, coronary heart diseases, coronary disease, ischemic heart disease, myocardial ischemia, heart failure, cardiac failure, myocardial infarction, myocardial disease, cardiometabolic disease, cardiovascular surgery, percutaneous intervention, percutaneous transluminal coronary angioplasty, coronary angioplasty);

Intervention (multitasking behavior, dual task, dual task training, dual task intervention, dual task exercise, cognitive motor, cognitive motor, cognitive motor training, motor cognitive training, multimodal training, multimodal exercise, cognitive motor exercise, mind motor, dual tasking, dual task, multitasking, exergaming, virtual reality, exergame, exergames, kinect based exergaming, virtual reality exercises, virtual reality training, decision making, brain, cognitive training, cognitive exercise, brain training, brain exercise, rehabilitation exercise, physical therapy, physical training, aerobic training, anaerobic training, circuit based training, physical exercise, physical fitness, physical exertion, effort, home based exercise, aerobic exercise, anaerobic exercise, circuit based exercise, exercise therapy, cardiac rehabilitation, mobility, walking);

Outcome (Cognition, cognitive function, EF, attention, memory functions, working memory, inhibitory control, verbal fluency, cognitive impairment, mild cognitive impairment, cognitive dysfunction, cognitive decline, cognitive performance, decision making, spatial navigation, brain activation, neurocognition, Brain-Derived Neurotrophic Factor, BDNF, visuospatial ability, cognitive motor interference, cognitive performance).
